# TIME TO ENHANCE THE MESSAGE? KNOWLEDGE OF DEMENTIA RISK AND SYMPTOMS IN CENTRAL NORTH CAROLINA

**DOI:** 10.1093/geroni/igae098.3756

**Published:** 2024-12-31

**Authors:** Cassandra Germain, Travonia Brown-Huges

**Affiliations:** North Carolina A&T State Univeristy, Greensboro, North Carolina, United States; North Carolina A&T State University, Greensboro, North Carolina, United States

## Abstract

It is well known that Black-American and Latino adults are at higher risk for Alzheimer’s disease compared to their White counterparts. Yet, misconceptions about AD persist in ethnic-minority communities. For example, a recent Alzheimer’s Association Report (2021) suggests that concern about developing Alzheimer’s is lower among Native Americans (25%), Blacks (35%) and Hispanics (41%), compared with Whites (48%) and more than half of non-White Americans believe significant loss of memory or cognitive abilities is a normal part of aging. The current data is part of an ongoing study designed to evaluate existing knowledge about dementia risk factors and the early warning signs of dementia in low-moderate income communities in Central North Carolina. A 17-item true/false questionnaire framed as a “Brain Health Trivia” quiz was used to assess knowledge. Participants had the choice of completing an electronic (cell phone or tablet) or paper version. The electronic version provided respondents with an immediate completion score as well as the correct answers to incorrect questions. To date (N=307) adults aged 18+ have completed the survey. Results show that community members are knowledgeable about many preventive factors such as the importance of diet and exercise, but are less knowledgeable about the impact of chronic disease on brain health. Nearly 45% of respondents surveyed were not aware that diabetes was a risk factor for dementia and 34.4% were not aware that hypertension was a risk factor. These results demonstrate the need for ongoing education and outreach efforts on brain health particularly in low-moderately resourced communities.

